# Correction: The effects of assistance dogs on psychosocial health and wellbeing: A systematic literature review

**DOI:** 10.1371/journal.pone.0256071

**Published:** 2021-08-09

**Authors:** Kerri E. Rodriguez, Jamie Greer, Jane K. Yatcilla, Alan M. Beck, Marguerite E. O’Haire

The Funding statement is incorrect. The correct statement is: Funding to support open access publication of this manuscript was provided by The Society for Companion Animal Studies (SCAS).
