# Supplementary material for: The effects of assistance dogs on psychosocial health and wellbeing: A systematic literature review
Source: PLoS One. 2020 Dec 2;15(12):e0243302. doi: 10.1371/journal.pone.0243302 (PMC7710121; doi:10.1371/journal.pone.0243302)
Supplement: S1 Table — The search strategy was adapted to the other databases, including mapping terms to each database’s thesaurus or prescribed vocabulary, as appropriate. (DOCX) [file pone.0243302.s002.docx]

| **PubMed Search Strategy** |
| --- |
| ( “Service animal”[Title/Abstract] OR “service animals”[Title/Abstract] OR “Service dog”[Title/Abstract] OR “Service dogs”[Title/Abstract] OR “Assistance animal”[Title/Abstract] OR “Assistance animals”[Title/Abstract] OR “Assistance dog”[Title/Abstract] OR “Assistance dogs”[Title/Abstract] OR “Guide dog”[Title/Abstract] OR “Guide dogs”[Title/Abstract] OR “Dog guide”[Title/Abstract] OR “Dog guides”[Title/Abstract] OR “Mobility dog”[Title/Abstract] OR “Mobility dogs”[Title/Abstract] OR “Seizure dog”[Title/Abstract] OR “Seizure dogs”[Title/Abstract] OR “Seizure alert dog”[Title/Abstract] OR “Seizure alert dogs”[Title/Abstract] OR “Seizure response dog”[Title/Abstract] OR “Seizure response dogs”[Title/Abstract] OR “Epilepsy alert dog”[Title/Abstract] OR “Epilepsy alert dogs”[Title/Abstract] OR “Diabetes alert dog”[Title/Abstract] OR “Diabetes alert dogs”[Title/Abstract] OR “Diabetic alert dog”[Title/Abstract] OR “Diabetic alert dogs”[Title/Abstract] OR “Diabetic response dog”[Title/Abstract] OR “Diabetic response dogs”[Title/Abstract] OR “Hearing dog”[Title/Abstract] OR “Hearing dogs”[Title/Abstract] OR “Signal dog”[Title/Abstract] OR “Signal dogs”[Title/Abstract] OR “Medical response dog”[Title/Abstract] OR “Medical response dogs”[Title/Abstract] OR “Seeing eye dog”[Title/Abstract] OR “Seeing eye dogs”[Title/Abstract] ) |
