# Supplementary material for: The effects of assistance dogs on psychosocial health and wellbeing: A systematic literature review
Source: PLoS One. 2020 Dec 2;15(12):e0243302. doi: 10.1371/journal.pone.0243302 (PMC7710121; doi:10.1371/journal.pone.0243302)
Supplement: S2 Table — Studies are organized by design (longitudinal or cross-sectional). (DOCX) [file pone.0243302.s003.docx]

| Study Design | First Author (Year) | Study # | Objective | Hypothesis | Demographics | Disabilities | Equal groups | Inclusion/exclusion | | Ethical approval | | Service dog characteristics | | Control | | Variability estimates | Statistical values | Effect sizes | Precise probability values | Service dog time | Limitations | Total | | % |
| --- | --- | --- | --- | --- | --- | --- | --- | --- | --- | --- | --- | --- | --- | --- | --- | --- | --- | --- | --- | --- | --- | --- | --- | --- |
| Longitudinal | Allen (1996) | 1 | 1 | 0 | 1 | 1 | 1 | 1 | | 0 | | 0 | | 1 | | 1 | 0 | 0 | 1 | - | 1 | 9/14 | | 64% |
|  | Collins (2004) | 2 | 1 | 1 | 1 | 1 | 1 | 1 | | 1 | | 0 | | 1 | | 1 | 0 | 0 | 0 | - | 1 | 10/14 | | 71% |
|  | Donovan (1994) | 3 | 1 | 1 | 1 | 1 | 1 | 0 | | 1 | | 0 | | 1 | | 0 | 0 | 0 | 0 | - | 1 | 8/14 | | 57% |
|  | Gilbey (2003) #1 | 4 | 1 | 1 | 1 | 0 | - | 0 | | 0 | | 0 | | 0 | | 0 | 1 | 0 | 1 | - | 1 | 6/13 | | 46% |
|  | Guest (2006) | 5 | 1 | 0 | 1 | 1 | - | 0 | | 0 | | 0 | | 0 | | 0 | 0 | 0 | 0 | - | 0 | 3/13 | | 23% |
|  | Hubert (2013) | 6 | 1 | 0 | 1 | 1 | - | 1 | | 1 | | 0 | | 0 | | 1 | 0 | 0 | 1 | - | 1 | 8/13 | | 62% |
|  | Lundqvist (2018) | 7 | 1 | 0 | 1 | 1 | - | 1 | | 1 | | 1 | | 0 | | 1 | 0 | 1 | 1 | - | 1 | 10/13 | | 77% |
|  | Rabschutz (2006) | 8 | 1 | 0 | 1 | 1 | - | 0 | | 1 | | 1 | | 0 | | 1 | 0 | 0 | 0 | - | 1 | 7/13 | | 54% |
|  | Rintala (2008) #1 | 9 | 1 | 1 | 1 | 1 | 1 | 1 | | 0 | | 0 | | 1 | | 1 | 1 | 0 | 1 | - | 1 | 11/14 | | 79% |
|  | Rintala (2008) #2 | 10 | 1 | 1 | 1 | 1 | 0 | 1 | | 0 | | 0 | | 1 | | 1 | 1 | 0 | 1 | - | 1 | 10/14 | | 71% |
|  | Spence (2015) | 11 | 1 | 0 | 1 | 1 | 0 | 0 | | 1 | | 1 | | 1 | | 0 | 0 | 0 | 0 | - | 0 | 6/14 | | 43% |
|  | Vincent (2017) | 12 | 1 | 1 | 1 | 1 | - | 0 | | 1 | | 1 | | 0 | | 1 | 0 | 0 | 1 | - | 1 | 9/13 | | 69% |
| Cross-sectional | Collins (2006) | 13 | 1 | 1 | 1 | 1 | 1 | 1 | | 0 | | 0 | | 1 | | 1 | 0 | 0 | 0 | 1 | 1 | 10/15 | | 67% |
|  | Craft (2007) | 14 | 1 | 1 | 1 | 0 | 0 | 1 | | 0 | | 0 | | 1 | | 1 | 1 | 0 | 1 | 0 | 1 | 9/15 | | 60% |
|  | Crudden (2017) | 15 | 1 | 0 | 0 | 1 | 1 | 1 | | 1 | | 0 | | 1 | | 1 | 1 | 1 | 0 | 0 | 1 | 10/15 | | 67% |
|  | Davis (2017) | 16 | 1 | 1 | 1 | 1 | 0 | 1 | | 1 | | 0 | | 1 | | 1 | 1 | 1 | 1 | 0 | 1 | 12/15 | | 80% |
|  | Gilbey (2003) #2 | 17 | 1 | 1 | 1 | 0 | 1 | 0 | | 0 | | 0 | | 1 | | 1 | 1 | 0 | 1 | 0 | 1 | 9/15 | | 60% |
|  | Hacket (1994) | 18 | 1 | 0 | 1 | 1 | 1 | 1 | | 1 | | 0 | | 1 | | 0 | 1 | 0 | 1 | 1 | 1 | 11/15 | | 73% |
|  | Hall (2017) #1 | 19 | 1 | 1 | 0 | 1 | 1 | 1 | | 1 | | 0 | | 1 | | 1 | 1 | 1 | 1 | 0 | 1 | 12/15 | | 80% |
|  | Hall (2017) #2 | 20 | 1 | 1 | 0 | 1 | 1 | 1 | | 1 | | 0 | | 1 | | 1 | 1 | 1 | 1 | 0 | 1 | 12/15 | | 80% |
|  | Matsunaka (2008) | 21 | 1 | 1 | 1 | 0 | 0 | 0 | | 1 | | 0 | | 1 | | 1 | 0 | 0 | 0 | 0 | 0 | 6/15 | | 40% |
|  | Milan (2007) | 22 | 1 | 1 | 1 | 1 | 1 | 1 | | 1 | | 0 | | 1 | | 1 | 0 | 0 | 0 | 0 | 1 | 10/15 | | 67% |
|  | Refson (1999) | 23 | 1 | 0 | 1 | 0 | 1 | 1 | | 0 | | 0 | | 1 | | 0 | 0 | 0 | 1 | 1 | 0 | 7/15 | | 47% |
|  | Rodriguez (2018) | 24 | 1 | 1 | 1 | 1 | 1 | 1 | | 1 | | 1 | | 1 | | 1 | 1 | 1 | 1 | 1 | 1 | 15/15 | | 100% |
|  | Rushing (1994) | 25 | 1 | 1 | 1 | 1 | 1 | 0 | | 0 | | 0 | | 1 | | 1 | 0 | 0 | 0 | 0 | 1 | 8/15 | | 53% |
|  | Shintani (2010) | 26 | 1 | 0 | 1 | 1 | 1 | 1 | | 0 | | 0 | | 1 | | 1 | 0 | 0 | 0 | 0 | 0 | 7/15 | | 47% |
|  | Yarmolkevich (2017) | 27 | 1 | 1 | 0 | 1 | 0 | 0 | | 1 | | 0 | | 1 | | 1 | 1 | 0 | 0 | 0 | 1 | 8/15 | | 53% |
| Total Yes | | | 27 | 17 | 23 | 22 | 15 | 17 | 16 | | 5 | | 21 | | 21 | | 12 | 6 | 15 | 4 | 22 |  |  | |
| Total No | | | 0 | 10 | 4 | 5 | 6 | 10 | 11 | | 22 | | 6 | | 6 | | 15 | 21 | 12 | 11 | 5 |  |  | |
| Total N/A | | | 0 | 0 | 0 | 0 | 6 | 0 | 0 | | 0 | | 0 | | 0 | | 0 | 0 | 0 | 12 | 0 |  |  | |
