# Supplementary material for: The effects of assistance dogs on psychosocial health and wellbeing: A systematic literature review
Source: PLoS One. 2020 Dec 2;15(12):e0243302. doi: 10.1371/journal.pone.0243302 (PMC7710121; doi:10.1371/journal.pone.0243302)
Supplement: Attachment — Submitted filename: ResponseToReviewers.docx [file pone.0243302.s004.docx]

# Editor Comments

1. Please ensure that your manuscript meets PLOS ONE's style requirements, including those for file naming. The PLOS ONE style templates can be found at https://journals.plos.org/plosone/s/file?id=wjVg/PLOSOne_formatting_sample_main_body.pdf and https://journals.plos.org/plosone/s/file?id=ba62/PLOSOne_formatting_sample_title_authors_affiliations.pdf

We have fixed formatting on all tables, figures, and table/figure captions. We have also updated the title page formatting.

2. Please include captions for your Supporting Information files at the end of your manuscript, and update any in-text citations to match accordingly. Please see our Supporting Information guidelines for more information: [http://journals.plos.org/plosone/s/supporting-information](about:blank).

We have provided the supporting information captions at the bottom of the manuscript as requested, and updated all in-text citations for the S1 and S2 tables.

# Reviewer 1

1-Regarding Research Question 1: researchers should be consistent in using terms. Problem that they used different terms: Sometime describe, identify, another time, summarize for question 1.
Authors should determine the appropriate terms for three questions, keeping using the same terms in the whole paper.

We have edited the text throughout the paper such that each of the three aims are consistent with the following wording: “The specific aims were to (1) describe the key characteristics of studies (2) evaluate the methodological rigor of studies (3) summarize outcomes.”

2-In Discussion Section, authors are clear in mentioning the third aim. I don’t read or see: first aim and second aim. Authors should be add it

Thank you for this suggestion. In addition to the headers stating each aim, we also start each section with the appropriate language e.g. “Our second aim was to evaluate the methodological rigor of studies. We found that…”

3 -Based on my experiences for teaching the systematic review, systematic researchers should provide their manuscript by official or scientific paragraph terms containing terminologies of subjects of systematic reviews that they use it in search database. Why did not researchers mention it at their article? All readers prefer to read and see that paragraph / terms used in journals. Example (Assistance or support or helping Dogs and ….etc.)

We provided the full MEDLINE search strategy in S1 Table, rather than in the text, so that the search strategy could be fully replicated by other researchers. We describe this in lines 167-169: “The complete MEDLINE search strategy, which was adapted for the other databases, is shown in S1 Table.” The table describes the full search terms used, as follows:

( “Service animal”[Title/Abstract] OR “service animals”[Title/Abstract] OR “Service dog”[Title/Abstract] OR “Service dogs”[Title/Abstract] OR “Assistance animal”[Title/Abstract] OR “Assistance animals”[Title/Abstract] OR “Assistance dog”[Title/Abstract] OR “Assistance dogs”[Title/Abstract] OR “Guide dog”[Title/Abstract] OR “Guide dogs”[Title/Abstract] OR “Dog guide”[Title/Abstract] OR “Dog guides”[Title/Abstract] OR “Mobility dog”[Title/Abstract] OR “Mobility dogs”[Title/Abstract] OR “Seizure dog”[Title/Abstract] OR “Seizure dogs”[Title/Abstract] OR “Seizure alert dog”[Title/Abstract] OR “Seizure alert dogs”[Title/Abstract] OR “Seizure response dog”[Title/Abstract] OR “Seizure response dogs”[Title/Abstract] OR “Epilepsy alert dog”[Title/Abstract] OR “Epilepsy alert dogs”[Title/Abstract] OR “Diabetes alert dog”[Title/Abstract] OR “Diabetes alert dogs”[Title/Abstract] OR “Diabetic alert dog”[Title/Abstract] OR “Diabetic alert dogs”[Title/Abstract] OR “Diabetic response dog”[Title/Abstract] OR “Diabetic response dogs”[Title/Abstract] OR “Hearing dog”[Title/Abstract] OR “Hearing dogs”[Title/Abstract] OR “Signal dog”[Title/Abstract] OR “Signal dogs”[Title/Abstract] OR “Medical response dog”[Title/Abstract] OR “Medical response dogs”[Title/Abstract] OR “Seeing eye dog”[Title/Abstract] OR “Seeing eye dogs”[Title/Abstract] )

4-(e.g. 0.035 rather than <0.05) why did researchers choose 0.035 as criterion? In Table 2. Summary of methodological ratings for N=27 studies … etc.

We apologize for this confusion. The method scoring item was “Have actual probability values been reported for most outcomes?” Using e.g. as the abbreviation for the Latin phrase meaning “for example”, we provided 0.035 as an example of exact probability reporting (rather than reporting <0.05). So, 0.035 was not used as any specific criterion, but rather as an example. We revised the text to say “e.g. reporting 0.035 rather than reporting <0.05” to clarify.

The subject of paper is viable, novelty and interested. Systematic review is hard working as I see that in this paper. So, in my opinion paper is acceptable with modification.

We thank the reviewer for their time and consideration reviewing this manuscript.

# Reviewer 2

This article is considering and dealing with an excellent subject which is not only sensitive but also helpful. The authors have thought and looked into the psychological benefits of guide dogs and the importance of a holistic approach on such issues. I am hoping by publishing this article, we would expect to see further research work from the same authors and others.

We thank the reviewer for their positive comments and time reviewing this manuscript.
